# Development of a Luliconazole Nanoemulsion as a Prospective Ophthalmic Delivery System for the Treatment of Fungal Keratitis: In Vitro and In Vivo Evaluation

**DOI:** 10.3390/pharmaceutics14102052

**Published:** 2022-09-26

**Authors:** Jingjing Yang, Zhen Liang, Ping Lu, Fei Song, Zhen Zhang, Tianyang Zhou, Jingguo Li, Junjie Zhang

**Affiliations:** Henan Eye Hospital, Henan Provincial People’s Hospital, People’s Hospital of Zhengzhou University, No. 7 Weiwu Road, Zhengzhou 450003, China

**Keywords:** luliconazole, nanoemulsion, ocular drug delivery, fungal keratitis, pharmacokinetics

## Abstract

Luliconazole (LCZ), a novel imidazole drug, has broad-spectrum and potential antifungal effects, which makes it a possible cure for fungal keratitis; nevertheless, its medical use in ocular infections is hindered by its poor solubility. The purpose of this study was to design and optimize LCZ nanoemulsion (LCZ-NE) formulations using the central composite design-response surface methodology, and to investigate its potential in improving bioavailability following ocular topical administration. The LCZ-NE formulation was composed of Capryol 90, ethoxylated hydrogenated castor oil, Transcutol^®^ P and water. The shape of LCZ-NE was spherical and uniform, with a droplet size of 18.43 ± 0.05 nm and a low polydispersity index (0.070 ± 0.008). The results of an in vitro release of LCZ study demonstrated that the LCZ-NE released more drug than an LCZ suspension (LCZ-Susp). Increases in the inhibition zone indicated that the in vitro antifungal activity of the LCZ-NE was significantly improved. An ocular irritation evaluation in rabbits showed that the LCZ-NE had a good tolerance in rabbit eyes. Ocular pharmacokinetics analysis revealed improved bioavailability in whole eye tissues that were treated with LCZ-NE, compared with those treated with LCZ-Susp. In conclusion, the optimized LCZ-NE formulation exhibited excellent physicochemical properties, good tolerance, enhanced antifungal activity and bioavailability in eyes. This formulation would be safe, and shows promise in effectively treating ocular fungal infections.

## 1. Introduction

Fungal keratitis (FK), a severe corneal infection, can lead to stromal destruction, ulceration, perforation, even endophthalmitis and corneal scar formation [1,2]. It is a serious sight-threatening disease which can cause permanent blindness, and even eyeball loss [3,4]. FK can be caused by more than one hundred different species; the main pathogenic fungi are filamentous fungi, e.g., *Fusarium* spp. and *Aspergillus* spp., and the yeast *Candida* spp. Most fungal eye infections in tropical and subtropical climates are filamentous fungi, while yeast infection is more frequent in temperate climates [5,6,7]. FK presents clinically corneal infiltrates with feathery-like edges, lifted surface, even deeply involved stroma with intact epithelium, and endothelial plaques [8]; some patients also develop endophthalmitis that requires evisceration, although advancements in antifungal agents and surgical technologies have improved therapies [9,10,11]. FK is poorly treated, and has a high morbidity. It is still a challenge for ophthalmologists and pharmaceutical researchers to screen for effective drugs and ocular delivery systems which could penetrate the cornea or other intraocular barriers.

Topical instillation with an antifungal drug is the first treatment choice to for FK. Natamycin is considered the mainstay of treatment for FK, and is the only available approved ocular application used as this indication by the Food and Drug Administration in the United States [12,13,14]. However, the treatment of FK is still difficult, due to poor corneal penetration and the long course of therapy with natamycin suspensions; about 40% of patients with fungal keratitis also require keratoplasty or other treatment [5,15]. Therefore, there is an urgent need to develop more effective drugs. Luliconazole (LCZ) is a newly added imidazole antifungal agent, which has widely antifungal activity against pathogenic fungi, especially including *Fusarium, Aspergillus* and yeasts that cause most ocular fungal diseases [16,17,18]. The clinical effectiveness of LCZ is limited by its poor aqueous solubility (approximately 0.065 mg/mL at 25 °C), which decreases its bioavailability [19]. Compared with other azoles, LCZ shows 1–4-fold smaller minimum inhibitory concentrations (MIC) than many other antifungal drug, and still exhibits activity against strains which have resistance to fluconazole [20]. Therefore, it is necessary to formulate LCZ into an ocular drug delivery system that can deliver it into the eye, in order to achieve therapeutic effects after topical administration.

The most ideal method of administration for treatment of ocular infection is to instill topically into the eye, assuming ease of handling, patient compliance and low costs. With the eye’s unique anatomy and physiology, it is difficult for the drug to reach the target site of action; only less 3% of the administered dosage penetrates the corneal barrier into intraocular tissues [21,22]. These barriers limit ocular absorption, thus decreasing the bioavailability of common eyedrop formulations (e.g., solutions and suspensions). Therefore, it is very important for ocular drug delivery systems (ODDS) to sufficiently sustain precorneal retention time, in order to pass the barriers and increase ocular bioavailability after topical application [23]. In recent decades, various novel nanoscale carriers used as ODDS [24,25,26], e.g., liposomes, nanoemulsions (NEs), micelles, nanoparticles and in-situ gels, have been introduced as novel strategies to enhance intraocular penetration and ocular bioavailability. NEs, as ocular applications, offer ease of application, less frequent instillation and enhanced ocular bioavailability. Currently the nanoformulations or nanostructure carriers for luliconazole include nanoemulgel [27], spanlastic nanocarriers [28,29], nanostructured lipid carriers (NLCs) [30,31], niosomal gel-based systems [32], luliconazole-based SLN gel for vaginal infection [33], lyotropic liquid crystalline nanoparticles [34], vesicular-based gel formulations [19] and nanocrystal-loaded hydrogels [35]. These studies about the delivery of luliconazole mainly focused on cutaneous application or vaginal application, and the results demonstrated improved cutaneous permeation. Thus far, however, no investigations on luliconazole nanocarriers or NEs for ocular application have been reported.

Therefore, this research was aimed at developing an NE formulation of LCZ and optimizing it for drug delivery into the eye, in order to enhance the bioavailability of LCZ in the eye. Pseudoternary phase diagrams (PTPDs) were constructed to obtain the suitable ratio of surfactant and co-surfactant, and further used to optimize the LCZ nanoemulsion (LCZ-NE) by means of the central composite design-response surface methodology (CCD-RSM). LCZ-NE of the optimized formulation was made and characterized through droplet size (DS), ζ-potential (ZP), morphology, entrapment efficiency (EE) and in vitro drug release. Ocular irritation tests were conducted to observe tolerance of the developed formulation. Furthermore, the obtained LCZ-NE was tested in in vitro antifungal activity; the ocular pharmacokinetics as well as intraocular tissue distributions of the LCZ-NE were evaluated in rabbit eyes, post-ocular application.

## 2. Materials and Methods

### 2.1. Materials

LCZ was purchased from Macklin Biochemical Co., Ltd. (Shanghai, China); oleic acid was obtained from Tokyo Chemical Industries Co., Ltd. (Tokyo, Japan); propylene glycol dicaprylate (PGD) and castor oil were obtained from Hunan Erkang Pharmaceutical Co., Ltd. (Hunan Province, Changsha, China); Cremophor^®^ RH (RH40), Cremophor^®^ EL35 (EL35) and Kolliphor^®^ HS15 (HS15) were obtained from BASF SE (Ludwigshafen, Germany); Tween 80 was obtained from Jinshan Pharmaceutical Co., Ltd. (Sichuan Province, Meishan, China); polyethylene glycol 400 (PEG400) was purchased from Solarbio Life Science (Beijing, China); Capryol 90, Lauroglycol™ FCC, Lauroglycol and Transcutol® P were received as gifts from Gattefosse (Lyon, France). Methanol and acetonitrile (HPLC grade) were obtained from Merck (Darmstadt, Germany) and Tedia (Fairfield, OH, USA), respectively. Other reagents (analytical grade) were purchased from commercial suppliers.

### 2.2. Animals

New Zealand white rabbits (with a weight of 2.0 to 2.5 kg each) were used in the in vivo studies (provided by Huaxing Experiment Animal Breeding Co. (Zhengzhou, China)). All animal protocols were approved by the Ethical Committee of Experimental Animal Care of Henan Eye Institute (approval No. HNEECA-2021-03), and were conducted following the guidelines of the National Institutes of Health and the Association for Research in Vision and Ophthalmology Resolution. The rabbits were housed in air-conditioned, light-controlled rooms with a humidity of 60 ± 5% and a temperature of 20 ± 5 ℃. All rabbits were provided with a standard rod-like diet and drinking water, and were fasted overnight before beginning the experiment. All of the animals were healthy, and were assessed to be free of ocular diseases via a check using a hand slit-lamp.

### 2.3. Determining the Solubility of LCZ in Oils, Surfactants and Co-Surfactants

The solubility of LCZ in different oils, such as oleic acid (OA), olive oil, PGD, Capryol 90 and triacetin, in surfactants including Lauroglycol FCC, Lauroglycol 90, EL 35, RH 40, HS 15 and Tween 80, in co-surfactants such as 1,2-propylene glycol, PEG 400 and Transcutol P, were measured at 37 ℃ ± 2 ℃. Excess quantities of LCZ were added in 1-gram quantities to the above-mentioned oils, surfactants and co-surfactants in glass bottles, and the mixtures were mixed and shaken in a thermostatic oscillator at a speed of 100 rpm for 72 h to achieve equilibrium. Mixtures in each bottle were transferred into Eppendorf vials, and then centrifuged at 12,000 rpm for 15 min to precipitate particles of drug. The amounts in the supernatants were assayed using HPLC, according to a previously reported method [36]. Briefly, the quantitative supernatants were diluted with methanol, and the solubility of LCZ in oils, surfactants and co-surfactants was assayed via the HPLC method equipped with an ultraviolet detector (Waters 2487) and an octadecyl bonded silica column (C18, Waters XBridge^®^, ϕ 4.6 mm × 150 mm, particle size: 3.5 μm) at a detection wavelength of 290 nm. The mobile phase was the mixture of acetonitrile and water (55:45, *v/v*), with a flow rate of 0.5 mL/min. The oils, surfactants and co-surfactants were diluted with methanol for blank controls. Each experiment was conducted in triplicate. The oils, surfactants and co-surfactants that had the highest solubility for LCZ were used for subsequent studies. 

### 2.4. Screening of Surfactant for Emulsification Ability

Due to the solubilities of LCZ in those surfactants that exhibited no significant difference, different surfactants were further screened according to their abilities to emulsify Capryol 90, based on a previously reported method [37,38]. In brief, 300 mg of Capryol 90 was added to the same quantity of surfactants (EL 35, RH 40, HS 15 or Tween 80). The mixtures were vortexed for homogenization. Each of the mixtures (50 mg) was weighed, then diluted to 50 mL with purified water in a glass bottle with a rubber stopper, which was then was turned upside down using the same numbers of bottle inversions for each, in order to obtain a homogenous emulsion. These samples were left standing for 2 h; then, each sample’s transmittance (T, %) was determined using a UV-spectrophotometer at a wavelength of 650 nm, with purified water being used as a blank. Each test was conducted in triplicate.

### 2.5. Construction of PTPDs

The phase inversion composition method (PIC) was used to figure out the existence region of NEs, and the PTPDs were constructed at 37 ℃. From the PTPDs, the relative amount of the oil and the ratio of surfactant to co-surfactant were selected [39,40]. The ratios of surfactant to co-surfactant (Km) were 1:1, 2:1, 3:1 and 4:1 Then, the mixtures were made in different ratios of oil to Km (1:9, 2:8, 3:7, 4:6, 5:5, 6:4, 7:3, 8:2 and 9:1), and mixed with gentle magnetic stirring. Purified water was dropwise added into the mixture solution until transparent NEs appeared. The PTPDs were made according to the ultimate percentages of quantities of water, oil and Km, in the same system by means of Origin Pro Software (Version 9.1, Northampton, MA, USA).

### 2.6. Optimization of LCZ-NE by CCD-RSM

In order to optimize the formulation of LCZ-NE, Design-Expert 8.0 software (Stat-Ease, Inc., Minneapolis, MN, USA) was used for the design of the experiments [41,42]. The above-mentioned experiment suggested that two factors, including the quantity of the selected oil and the Km, influenced the properties of the NE. The quantities of the oil (X_1_) were 10–30%, and the Km (X_2_) was 1–4, as independent variables Formulation optimization was conducted using Design Expert 8.0 software; the NE droplet size (Y_1_, DS) and the drug content (Y_2_, DC) were used as evaluation indices (dependent variables). According to the experimental design scheme, the oil, surfactant and co-surfactant in a total quantity of 1 g were mixed well in a beaker using gentle magnetic stirring in a water bath at 37 ℃ ± 2 ℃; then, an excess amount of LCZ was added into the mixtures, with purified water added dropwise into the mixture until the total volume was 10 mL. After standing overnight, excess LCZ was removed using centrifugation (10,000 rpm for 10 min), and the DS was measured using dynamic light scattering (DLS) (NanoZS90, Malvern Instruments, Malvern, UK). The DC of LCZ-NE was assayed using HPLC. 

### 2.7. Preparation and Characterization of LCZ-NE

#### 2.7.1. Preparation of LCZ-NE

The optimal formulation of LCZ-NE was prepared on the basis of the optimizing results. Briefly, the drug, oil phase, surfactant and co-surfactant were mixed using gentle magnetic stirring at 37 ℃ until the drug was entirely dissolved. Then, injection water was dropwise added into the mixed solution until a transparent NE solution appeared. In order to meet the needs of the eyedrop formulation, the pH value was determined and adjusted to 5.0–7.0 by the dropwise addition of 0.1 M sodium hydroxide solution after cooling to ambient temperature. The solution was then metered to volume by injection water, filtered by a Millex-GP syringe-driven filter unit (0.22 μm, Merck Millipore, Cork, Ireland) into a sterilized vial.

The LCZ suspension (LCZ-Susp) was obtained by simply dissolving an equal amount of LCZ in injection water, before being pulverized using ultrasonication to obtain the LCZ-Susp, which was used as a control in the in vitro drug release study and in another subsequent investigation.

#### 2.7.2. Determination of DS, Polydispersity Index (PDI) and ZP

The average DS, PDI and ZP of the LCZ-NE were assayed using a Malvern Zetasizer, where DLS was monitored at 25 °C and with an angle of 90° after suitable dilution. Each value in all of the results was represented as a mean of three measurements. 

#### 2.7.3. Measurements of DC and EE

The total DC was assayed using the above-mentioned HPLC method (see Section 2.3) after suitable dilution by methanol. The drug EE was measured via ultrafiltration; in brief, an ultra-centrifugal filter device was applied to separate free drugs from the mixed solutions using the following process: 4 mL of LCZ-NE were added to the upper cells of the filter device and centrifuged at 4000 rpm for 15 min (5810R, Eppendorf, Germany). The filtrates were diluted for HPLC analysis using methanol, then the weight of free drug was calculated. The EEs were calculated as shown in Equation (1):

(1) $$EE\left(\%\right)=\frac{W_{t}-W_{f}}{W_{t}}\times 100\%$$

where *W_t_* is the whole quantity of LCZ before centrifugation, and *W_f_* is the quantity of free LCZ in the filtrate.

#### 2.7.4. Morphology Observation of LCZ-NE 

The morphology characteristic of the LCZ-NE was observed using transmission electron microscopy (TEM) (Joel JEM 1230, Tokyo, Japan). The sample was prepared as follows: a drop of LCZ-NE after suitable dilution was mounted on a carbon-coated copper grid and left for 2 min, using a filter paper to remove excess liquid. The sample was then negatively stained with a drop of 2% phosphotungstic acid solution, and loaded into the TEM system for observation after the carbon grid was air-dried at room temperature.

#### 2.7.5. In Vitro LCZ Release Study 

The in vitro LCZ release from LCZ-NE was determined using the dialysis bag diffusion technique. The release medium was the artificial tear solution (ATS) which contained 0.1% (*w*/*v*) sodium dodecyl sulfate (SDS) (pH 7.4). Aliquots (1 mL) of the LCZ-NE were sealed in dialysis bags (cellulose membrane with a MW cut-off of 3500 Daltons), which were then put into 150 mL of release medium. The drug release test was conducted in an incubator that was rotated at 150 rpm, and at a temperature of 37 ± 0.1 °C (Changzhou Putian Instrument Manufacture Co. Ltd., Jiangsu Province, Changzhou, China). The aliquots (1 mL) of the release medium were removed, then replenished with the same volume of fresh release medium at predetermined time points. The LCZ-Susp was used as a control. Each test was conducted in triplicate. The quantities of LCZ released were assayed using HPLC (see Section 2.3). Concentrations of the samples and cumulative quantities of LCZ released were obtained by the LCZ calibration curve and Equation (2). The percentages of LCZ released from the LCZ-loading system were plotted vs. time.

(2) $$Q\;\left(\%\right)=\frac{C_{n}V+V_{i}\sum_{i=0}^{i=n}C_{i}}{W_{0}}\times 100\%$$

where *W*_0_ is the amount of LCZ in the LCZ-NE which was initially added into the dialysis bag; *C_n_* is the concentration of LCZ in the release medium at the corresponding time point; *V* is the initially added volume of release medium; *V_i_* is the sampling volume at time point *i*; and *C_i_* is the concentration of LCZ at time point *i*.

#### 2.7.6. Fourier Transform-Infrared (FT-IR) Spectra Studies

A FT-IR spectrometer (Alpha II, Bruker, Germany) was used to investigate LCZ, blank NE, the physical mixture between LCZ and blank NE and LCZ-NE, in order to ensure the compatibility between pure drug and optimized formulation. The analyses of LCZ, drug-free NE and LCZ-NE were performed using attenuated total reflection mode. The recording area of each spectrum was obtained from 4000 cm^−1^ to 500 cm^−1^, with a resolution of 4 cm^−1^.

### 2.8. Thermodynamic Stability and Storage Stability Study

In order to study the thermodynamic stability of the LCZ-NE, three freeze/thaw rounds were performed on freshly prepared LCZ-NE [43]. In Brief, the LCZ-NE was added into a transparent glass bottle and sealed, then stored in a refrigerator at −20 ℃ for 18 h. After that, the bottle was removed, kept at room temperature (25 ℃) for 18 h and returned to the refrigerator with the same previous temperature for another 18 h, in order to fulfill the second cycle. Similarly, the LCZ-NE sample was placed in a thermostatic container at 40 ℃ for 2 h, then returned to room temperature for 2 h; subsequent cycles followed the above process. The LCZ-NE against mechanical stress was tested using the following process: the formulation was centrifuged at 12,000 rpm for 15 min (MiniSpin^®^ Plus, Eppendorf, Germany). The experiment was replicated three times. The processed samples were inspected for any changes in appearance, such as oil-water stratification and LCZ precipitation. The thermodynamic stability was evaluated via visual observation.

Five milliliters of the LCZ-NE were added into a transparent glass bottle with a sealed cover, then kept at conditions of 4 °C/25% relative humidity (RH), then 25 °C/60% RH, and 40 °C/75% RH. The physicochemical parameters such as DS, PDI, ZP and DC were determined according to the methods described earlier (Section 2.7.2 and Section 2.7.3) after preparation (day 0), 0.5, 1 and 2 months.

### 2.9. In Vitro Antifungal Study

In vitro antifungal activities of the LCZ-NE and LCZ-Susp were evaluated on thirty *Fusarium* and twenty *Aspergillus* isolates from clinical separations, by means of the broth microdilution method of Clinical and Laboratory Standards Institute M38-A2. Blank NE and pure media were used as controls. MIC_90_ of the strains tested were measured using SPSS Statistics 26.0.

The agar plate diffusion method was used to test the Inhibition zone of the LCZ-NE against *Fusarium* and *Aspergillus* from clinical isolates [44]. Four wells of each plate (ϕ6 mm) were punched onto the agar plate using a cornea ring-drill, and 50 μL of LCZ-NE, LCZ-Susp, blank NE and voriconazole solution were placed into each well. After being diluted to appropriate concentrations with saline, the blank NE and voriconazole solutions served as negative control and positive control, respectively. Each well contained 10 ng of LCZ for *Fusarium*, and the positive control well contained 5 μg of voriconazole; meanwhile for *Aspergillus*, each well contained 5 ng of LCZ, and the positive control well contained 0.5 μg of voriconazole. The inoculated plates were placed into an incubator at 37 ℃ to culture for 24 h. The diameters of the inhibition zones were determined using a caliper.

### 2.10. In Vivo Ocular Irritation Study

Eye irritancy evaluation of the selected LCZ-NE eye drops were carried out and compared to the 0.9% saline solution. The ocular tolerance, the potential irritancy and the defects of LCZ-NE ophthalmic drops were tested using the modified scoring scale for eye irritation from the Draize test [45]. The right eyes of all six rabbits were chosen for testing purposes. Approximately 100 μL of LCZ-NE was dropped in the lower conjunctival sac, while contralateral (the left) eyes were instilled with the same amount of 0.9% NaCl as a negative control. The eyelids were softly closed for about 15 s, and Draize’s eye test was used to assess the ocular safety of the LCZ-NE eye drops. Any damage or signs and symptoms in the eyelids, cornea, iris and conjunctiva of bilateral eye photographs were recorded at 1, 2, 4, 24, 48 and 72 h, post application; an anterior segment camera system (Kanghua, Chongqing, China) was used to check and record under both normal and cobalt blue light, post-fluorescein-sodium instilled in the eyes, and scorings were carried out. The corneal opacity and iris hyperaemia severity were graded with scales that ranged from 0 to 4 and 0 to 2, respectively, and the congestion of the conjunctiva, edema of eyelids and discharge were marked on scales that ranged from 0–3, 0–4 and 0–3, respectively. The irritation severities were evaluated according to the total scores of all items that were based on the following ratings: no irritation, 0 to 3; slight irritation, 4 to 8; moderate irritation, 9 to 12; and severe irritation, 13 to 16.

### 2.11. In Vivo Ocular Pharmacokinetics in Rabbit

The healthy rabbits (42, each weighing 2 to 2.5 kg), without ocular diseases, were randomly assigned into a test group and a control group, with 21 animals in each group. Both eyes of every rabbit in the test group were instilled with one single dose of 50 μL of LCZ-NE, and the eyelids were closed for 15 s after application. Meanwhile, the control group animals were administered one single dose of 50 μL of LCZ-Susp. The rabbits were sacrificed at predetermined time points, post application, using an overdosage of phenobarbital sodium solution (4%, *w*/*v*) that was administered by injection through a marginal ear vein. The aqueous humor (AH) was removed using a sterile syringe with a 26-gauge needle from the limbus after the ocular surface was flushed with saline; then, the ocular tissues were immediately harvested and washed using saline, while the residual liquid was sucked up using filter paper. Then, the harvested conjunctiva, cornea, iris-ciliary body, lens, retinal choroid and sclera tissues were placed in precisely pre-weighed EP vials, which were then precisely weighed. Each AH or vitreous humor (VH) sample was precisely divided into aliquots of 100 μL. All biological samples were kept at −80 °C until assayed using a previously published HPLC-MS/MS method [46].

### 2.12. Statistical Analysis

All experimental data were expressed as means ± standard deviations (mean ± SD). An independent-sample t-test was used to evaluate the LCZ-NE and LCZ-Susp in vitro drug release and in vivo ocular pharmacokinetic studies with SPSS 26.0, where statistical significance was set at *p*-values < 0.05. Pharmacokinetic parameters were calculated by DAS 2.1.1 software (Anhui Provincial Center for Drug Clinical Evaluation, Wuhu, China).

## 3. Results and Discussion

### 3.1. Solubility of LCZ in Oils, Surfactants and Co-Surfactants

One of the key aspects of any successful solution formulation is its solubility. The selected solvent system must make LCZ solubilize above the desired level [47]. Thus, the solubility of LCZ in appropriate quantities of the NE excipients is a key factor in a successful formulation that maintains the drug in solution or colloidal form [48]. The drug loading capability of the oil phase is one of the most important considerations in the formulation of a NE. Higher drug solubility in the oil phase not only improves the drug loading capability, but also decreases the requirements of surfactant and co-surfactant, thereby minimizing their toxic effects [49]. 

**Figure 1:**
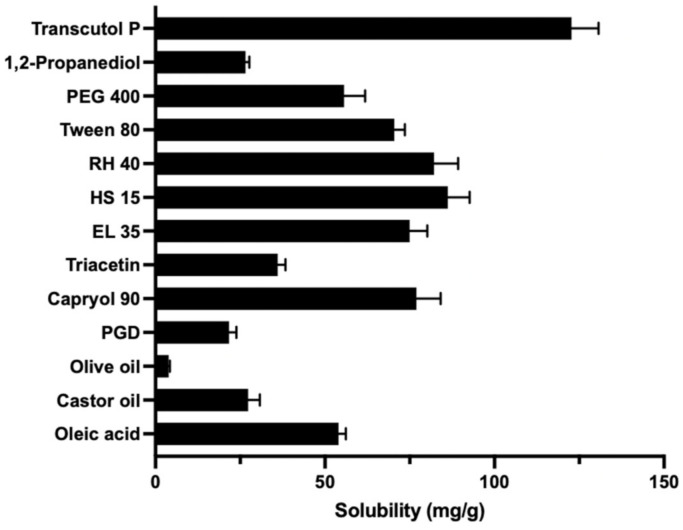
The solubility of LCZ in different oils, surfactants and co-surfactants (mean ± SD, *n* = 3).

Figure 1 exhibits the solubility of LCZ in different oils, surfactants and co-surfactants. The results suggested that the solubility of LCZ in Capryol 90 (76.96 ± 7.16 mg/g) was higher than in other oils, thus Capryol 90 was chosen as the oil solvent. The solubilities of LCZ in EL35, RH40, HS15 and Tween 80 were 74.98 ± 5.21 mg/g, 82.20 ± 7.05 mg/g, 86.20 ± 6.49 mg/g and 70.46 ± 3.13 mg/g, respectively; since these results were similar, their emulsifying abilities had to be determined in order to select an appropriate surfactant. Transcutol^®^ P demonstrated highest solubility for LCZ (122.73 ± 7.99 mg/g) among the tested co-surfactants, and it thus was used for next investigation. As Capryol 90 and Transcutol^®^ P have been widely used in many dosage forms, they have been licensed by European and US regulatory authorities for topical use, and thus recognized as safe [50]. 

### 3.2. Screening of Surfactant for Emulsifying Ability

**Figure 2:**
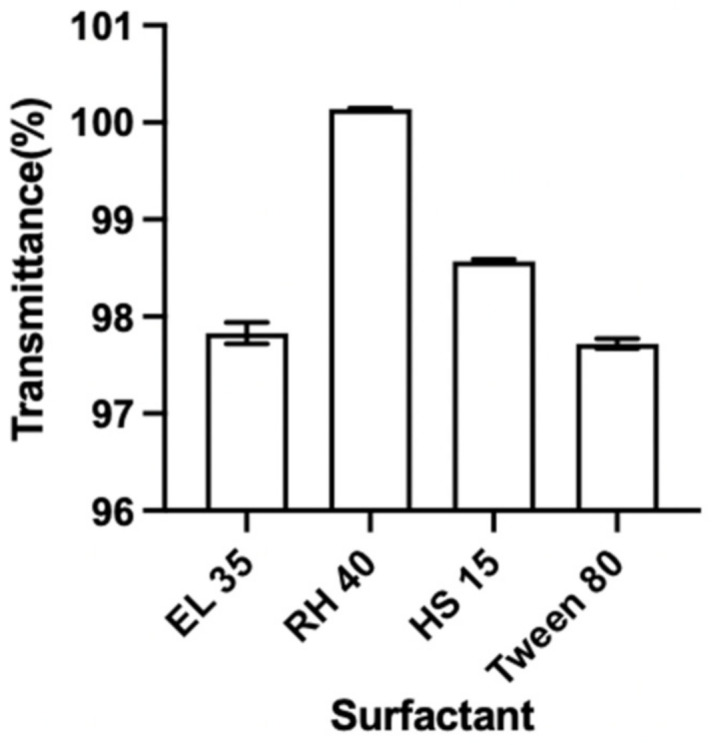
Transmittance (T%) of Capryol 90 with different surfactants (mean ± SD, *n* = 3).

A surfactant’s emulsifying ability is the key to it being selected [51]. The emulsifying ability of each surfactant to disperse Capryol 90 was evaluated by testing the T% of the resulting NE. High T% of the resulting systems indicated their optical clarity, and presented their nano size range (<100 nm), which could be caused by the high content of emulsifiers (50% *w*/*w*) [52]. The T% of each mixture composed of Capryol 90 and different surfactants was detected, and the results are shown in Figure 2. The results suggest that these surfactants exhibited very good emulsifying abilities to the selected oil. The T% of the mixtures that were composed of Capryol 90 and RH 40, EL 35, HS 15 and Tween 80, were 100.14 ± 0.01%, 97.83 ± 0.11%, 98.57 ± 0.02% and 97.72 ± 0.05%, respectively. Given surfactant structure, the fluidizing functional groups, e.g., double bonds or branches in the hydrophobic chain of the surfactant, enhanced the formation of the NE and enlarged the NE area in the PTPD; the functional groups make the surfactant more flexible to accept different curvatures [40]. Furthermore, RH 40 may enhance the penetration of the alkyl chain of the surfactant into the oil phase, thus improving the penetration of oil into the surfactant film [53]. Therefore, RH 40 was selected as the surfactant of NE for further study. 

### 3.3. Construction of PTPD

**Figure 3:**
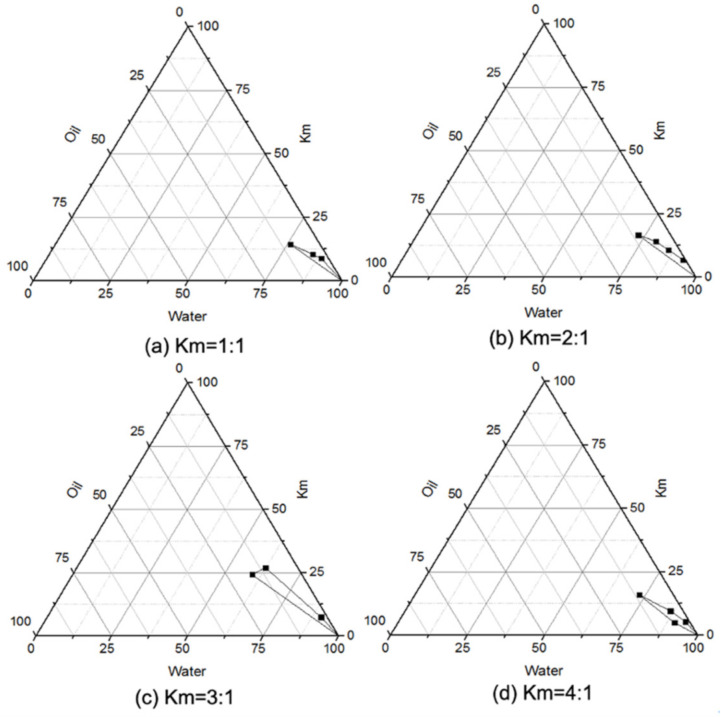
Pseudoternary phase diagrams consisting of Capryol 90, RH 40 and Transcutol® P, with Km values of (**a**) 1:1, (**b**) 2:1, (**c**) 3:1, (**d**) 4:1.

Constructing PTPDs is an effective means to investigate the phase behavior of a mixture of oil, surfactant, co-surfactant and water, as well as the effects of the compositions on the NE area [40]. The area from the transparent to translucent NE region in the investigated systems are illustrated with PTPDs, in which three sides (axes) of the largest triangle represent the percentage of water, oil and Km (at a fixed weight ratio of surfactant and co-surfactant) in the system. The rest of the field of the PTPD represents the turbid and common emulsion, according to visual appearance. The PTPDs of the NE consisted of Capryol 90 as the oil phase, RH 40 as the surfactant and Transcutol^®^ P as the co-surfactant, and are illustrated in Figure 3. The results indicate that the area of the NE field did not increase significantly with increasing Km. Therefore, the range of Km from 1 to 4 was used in the next CCD-RSM investigation.

### 3.4. Optimization of LCZ-NE by CCD-RSM

**Figure 4:**
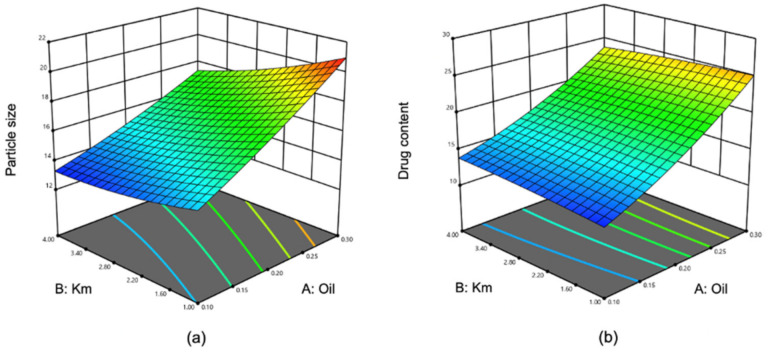
Three-dimensional response surface charts exhibiting the effects of independent variables (**a**) on DS (Y_1_) and (**b**) on DC (Y_2_).

**Figure 5:**
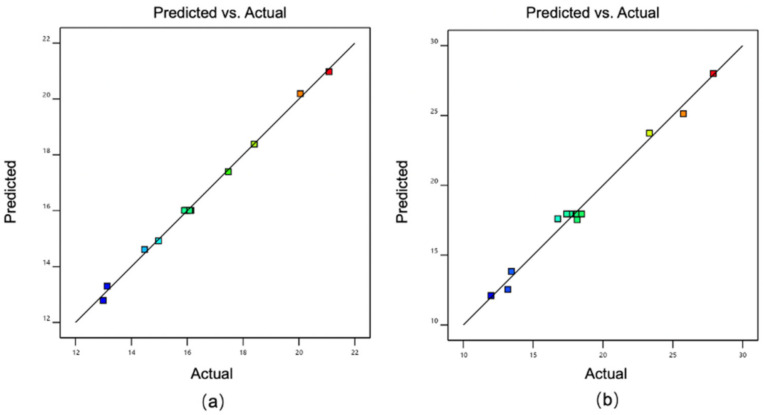
Actual values of DS and DC versus their predicted values (**a**): DS, (**b**): DC.

According to central composite design, 13 experiments were designed and completed, and the DS and DC of the NEs were assayed. The results are listed in Table 1. Then, the data were fitted to the models by means of RSM, and the three-dimensional response surface is displayed in Figure 4. The predicted values reasonably agreed with the actual values (Figure 5). The quadratic polynomial fitting equations that described the functional relationship between dependent variables (Y_1_: DS and Y_2_: DC) and independent variables (X_1_: weight of oil and X_2_: Km) are summarized as follows:

$$Y_{1}=12.77393+26.07627\;\times \;X_{1}-0.77395\;\times \;X_{2}-3.78333\;\times \;X_{1}X_{2}+23.87500\;\times \;{X_{1}}^{2}+0.14278\;\times \;{X_{2}}^{2}\;(R^{2}=0.9976).$$

$$Y_{2}=8.19633+25.26952\;\times \;X_{1}+1.29854\;\times \;X_{2}-4.46667\;\times \;X_{1}X_{2}+105.28750\;\times \;{X_{1}}^{2}-0.084278\;\times \;{X_{2}}^{2}\;(R^{2}=0.9889).$$

The squared of correlation coefficients (R^2^) of the quadratic polynomial fitting equations for DS and DC were 0.9976 and 0.9889, respectively, which suggested good fits of the model. 

As shown in Table 1, a low DS was obtained with the runs in which the percentage of oil (Capryol 90) was low while that of Km was high. This indicates that the DS tends to constrict and become stabilized with a max quantity of Km along with the percentage of oil decreasing. The lowest DS was 12.99 nm, while the highest DS was 21.08 nm. The broad range of DS on the design space demonstrated the effects of the selected independent variables on the DS. Instead, the DC is high when the percentage of Capryol 90 is at high level. The reason for this is because the increased amount of the oil enhances the LCZ that is solubilized in the system. The DS of an NE should be as small as possible, in order to penetrate deeper ocular tissues. The plot of DS (Figure 4a) proved that the oil proportion needs to be smaller in order to have the minimum DS of an NE. The chart of DC (Figure 4b) shows that oil proportion significantly influenced DC. It has been confirmed that retention of a drug depended on the distribution gradient of the drug between the vehicle of the NE and the eye tissues [54,55]. Thus, the composition of an NE that exhibits low DS and high DC was considered to be an ideal formulation.

### 3.5. Preparation and Characterization of LCZ-NE

#### 3.5.1. Preparation and Physicochemical Properties of LCZ-NE

**Figure 6:**
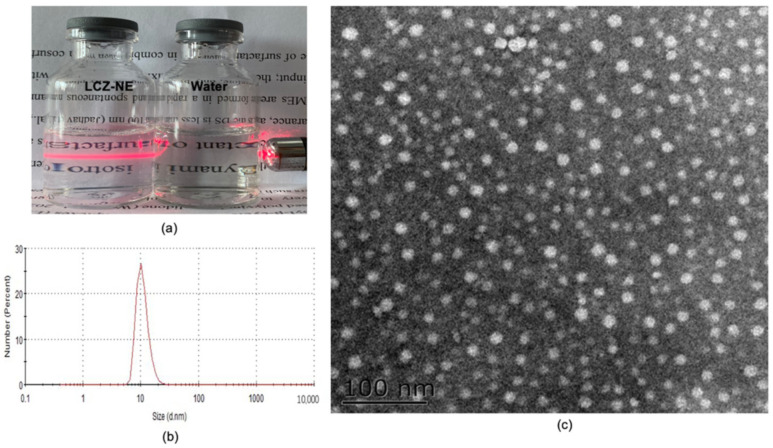
Appearance (**a**), size distribution (**b**) and TEM image (**c**) of the LCZ-NE. Scale bar = 100 nm.

According to the results of CCD-RSM, the optimized LCZ-NE formulation was composed of 0.2% LCZ, 3% Capryol 90, 5.5% RH 40 and 1.5% Transcutol^®^ P (*w*/*v*), with which the drug was soluble and could be loaded into the NE system. The appearance of LCZ-NE was visually clear, and with the Tyndall effect (Figure 6a). The physicochemical properties, including DS, PDI, ZP, EE and pH, are summarized in Table 2. The DS of the LCZ-NE was 18.43 ± 0.05 nm, as shown in Figure 6b, with a low PDI value of 0.070 ± 0.008 which indicated that the LCZ-NE had a narrow size distribution and was homogenous. The TEM image of the LCZ-NE (Figure 6c) demonstrated that the droplets were spherical and uniform in shape, which was in accordance with the DS distribution. The DS observed on TEM images was similar to that determined by the DLS method. The pH value of the LCZ-NE ophthalmic formulation was 6.22 ± 0.04, which met the requirements for eyedrops. The optimized LCZ-NE had a high EE (98.37 ± 0.47%).

#### 3.5.2. In Vitro LCZ Release of LCZ-NE

**Figure 7:**
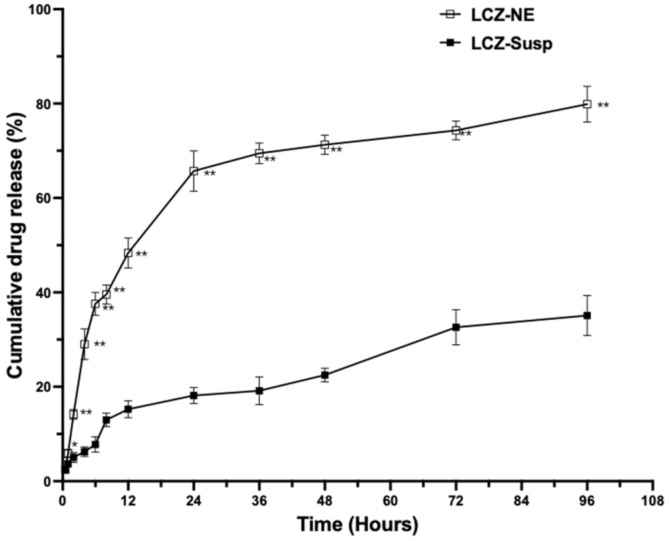
Drug release profile of LCZ from the LCZ-NE and LCZ-Susp. Data represented as mean ± SD, *n* = 3; * *p* < 0.05, ** *p* < 0.01, compared to LCZ-Susp.

The in vitro LCZ release behavior of the LCZ-NE and LCZ-Susp in artificial tear solution containing 0.1% SDS (*w*/*v*) was studied. As shown in Figure 7, almost 80% of LCZ was released from the NE formulation within 96 h; in comparison, only 35.10% of drug was released from the suspension. Except at the 0.5 h time point, the amount of cumulative LCZ release from the LCZ-NE was significantly higher than that from the LCZ-Susp (all *p* < 0.05) at all other tested time points. The observed results confirmed that the LCZ-NE could significantly enhance LCZ release compared to the pure drug suspension. The ability of NEs to increase the dissolution of poorly aqueous-soluble drugs has been observed in previous published research [43]. The in vitro release kinetics of LCZ from the LCZ-NE were analyzed using different mathematical models, with pure LCZ used as a control. The data of release were fitted using different equations, e.g., zero, first-order, Higuchi as well as Korsmeyer–Peppas equations, as displayed in Table 3. The first-order equation showed the best correlation coefficient (*R*^2^ = 0.9891) to the LCZ-NE, yet the LCZ-Susp was well fitted by the Higuchi equation (*R*^2^ = 0.9976). This model showed that the release of LCZ from the LCZ-NE implied that the drug release from NE was attributed to drug diffusion.

#### 3.5.3. FT-IR Studies

**Figure 8:**
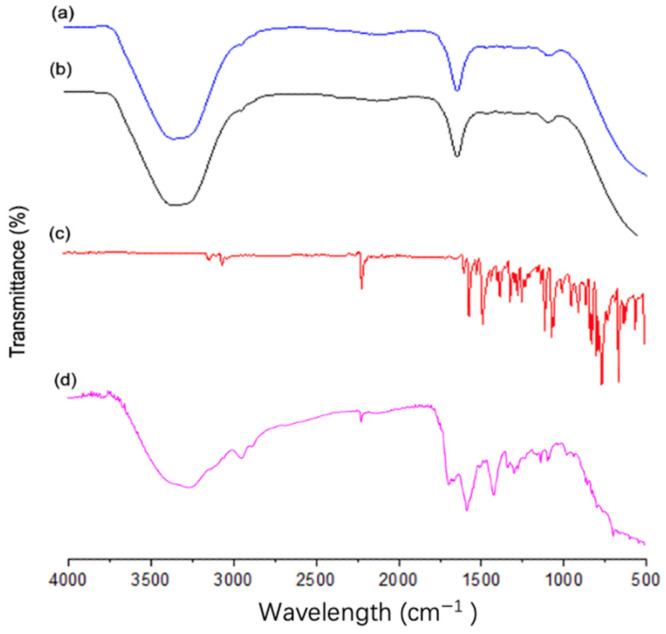
FT-IR spectra of LCZ-NE (**a**), blank NE (**b**), LCZ (**c**) and a physical mixture between LCZ and blank NE (**d**).

FT-IR was used to determine the formation of the LCZ-NE by means of analyzing the changes in the typical peak positions, shape and frequency. It is shown in Figure 8 that the FT-IR spectrum of pure LCZ displayed absorption peaks at 2196.92 cm^−1^ for C≡N stretching, 1699.29 cm^−1^ for C=N stretching, 1641.42 cm^−1^ for C=C alkene stretching, 1473 cm^−1^ for aromatic C=C stretching and 773.48 cm^−1^ for C-Cl bond stretching in the LCZ spectrum, which is in good agreement with previous literature [31]. However, the typical peaks of LCZ still existed in the FT-IR spectrum of the physical mixture between LCZ and blank NE, but disappeared in the spectrum of the LCZ-NE. The spectrum of the LCZ-NE was similar to that of the blank NE, with no new peaks occurring or potential shifts. The wide band at the region of 3300–3400 cm^−1^ was related to the water content in the formulation [56]. This result indicated that the disappearance of LCZ peaks in the NE could be due to complete solubilization or encapsulation of LCZ in Capryol 90 (oil phase). Chemical interactions between LCZ or polymer did not happen, thus this system may be stable [57,58]. Similar results were reported by Wan and coworkers on a tacrolimus-loaded microemulsion, and by Siafaka and coworkers with a brinzolamide-loaded microemulsion [58,59].

### 3.6. Stability Study of LCZ-NE

Another key aspect of any successful solution formulation is its stability. It is necessary to investigate the physicochemical properties of drug formulations, as they could be responsible for improving or reducing therapeutic efficiency [47]. An NE is a thermodynamically stable system of nano-scale size in which an immiscible liquid is dispersed in another, e.g., oil in water, with the help of a suitable surfactant and co-surfactant. In order to investigate the thermodynamic stability of the LCZ-NE, the capability of the formulation to maintain the original status was evaluated. It is a very important aspect to examine the appearance (e.g., clarity) of an NE, as the NE indicates instability and phase separation when the clear system become non-transparent [59]. In this study, the formulation did not display any signs of stratification, precipitation or turbidity after being processed by freezing, heating and high-speed centrifugation. This demonstrated that the formulation was physically stable under thermal and mechanical stress.

**Figure 9:**
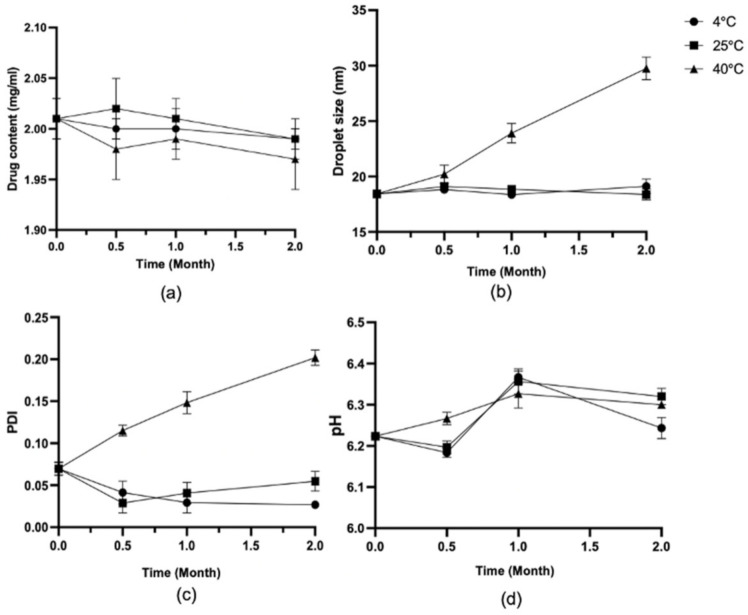
Changes in the LCZ-NE for DC (**a**), DS (**b**), PDI (**c**) and pH (**d**) under different conditions.

Samples of the LCZ-NE exhibited stable physical characteristics for 2 months after storage at 4 °C/25% RH and at 25 °C/60% RH, with no significant changes occurring in DS, PDI, pH value and DC, as shown in Figure 9. The formulation did not show precipitation or crystallization over the test period under both temperatures, which suggested that the LCZ-NE was relatively stable. However, both the DS and PDI showed an increasing trend at high temperature storage (40 °C/75% RH). At high temperature storage conditions, the DS varied from 18.43 ± 0.05 nm to 29.75 ± 1.02 nm, the PDI increased from 0.070 ± 0.007 to 0.202 ± 0.009, while the DC remained between 1.97 ± 0.03 mg/mL and 2.01 ± 0.02 mg/mL, and the pH value remained between 6.22 ± 0.006 and 6.33 ± 0.035. Perhaps the NE tended to coalesce at high temperature [60]. Thus, the LCZ-NE could be considered stable at 4 °C/25% RH and 25 °C/60% RH.

### 3.7. In Vitro Antifungal Study

**Figure 10:**
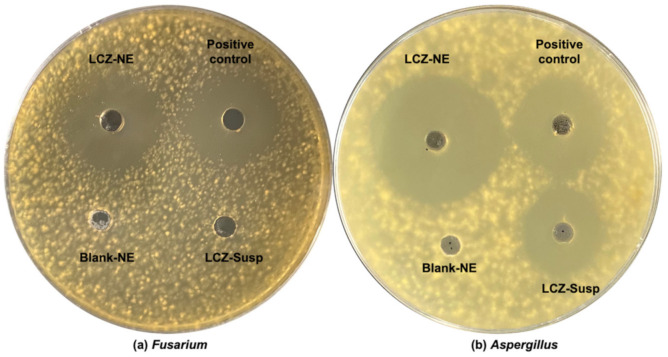
Antifungal activity of the LCZ-NE and LCZ-Susp against (**a**) *Fusarium* and (**b**) *Aspergillus* using the ager-plate diffusion method (cultured for 24 h under 37 °C).

The antifungal study revealed that LCZ possessed an enhanced antifungal effect when formulated in the NE form. The MIC_90_ values for LCZ against *Fusarium* and *Aspergillus* were decreased 2.3-fold and 2.5-fold, relative to the MIC_90_ values for LCZ-Susp, respectively (Table 4). Specifically, the MIC_90_ values for LCZ against *Fusarium* and *Aspergillus*, which were clinical isolates, were 10 and 2 ng/mL, respectively. The MIC_90_ values for the LCZ-Susp against *Fusarium* and *Aspergillus* were 23 and 5 ng/mL, respectively. The inhibition zones for the LCZ-NE increased relative to the LCZ-Susp when tested against *Fusarium* and *Aspergillus* clinical isolate. The diameters of the inhibition zones for the LCZ-NE against *Fusarium* and *Aspergillus* were 24.8 ± 1.10 mm and 35.4 ± 1.14 mm, respectively, while those for the LCZ-Susp against *Aspergillus* was 19.8 ± 0.84 mm. The inhibition zone could not be obtained for the LCZ-Susp against *Fusarium* clinical isolate treated at this dose (see Table 4 and Figure 10). The results indicate that the NE formulation enhanced the antifungal activity of LCZ compared to the suspension formulation.

### 3.8. In Vivo Ocular Irritation Study

**Figure 11:**
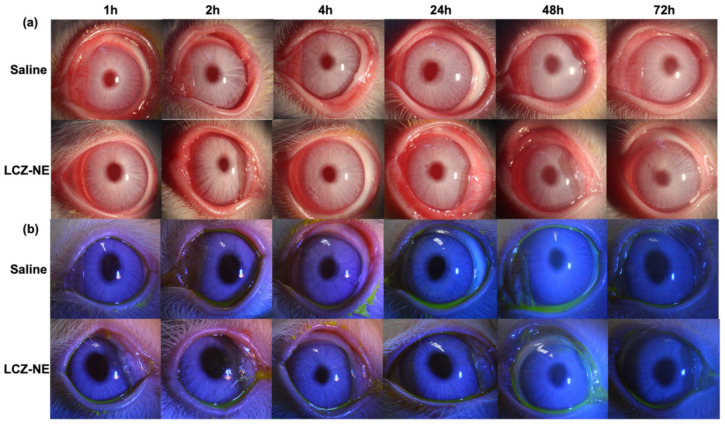
Ocular topical reaction observed by slit-lamp microscope after one application of the LCZ-NE and saline (as control) to rabbits. (**a**) Pictures with visible light and (**b**) cobalt blue light, post-fluorescein-sodium dropped into rabbit eyes.

After topical application of the LCZ-NE, there was no obvious irritation observed in the conjunctiva, cornea or iris of both the test and control eyes. The means of total scores were less than 1 at all observed time points, which met the conditions of no irritation. Sodium fluorescein solution was used to detect defects of the corneal epithelium, as observed under the slit-lamp microscope. There was no congestion, edema and discharge from the conjunctiva to the eye (Figure 11a), and there was no damage to the corneal epithelium observed by the slit-lamp microscope with cobalt blue light (Figure 11b), which indicated that the LCZ-NE caused no irritation to the eye. 

### 3.9. Ocular Pharmacokinetics and Tissues Distribution in Rabbit Eyes

**Figure 12:**
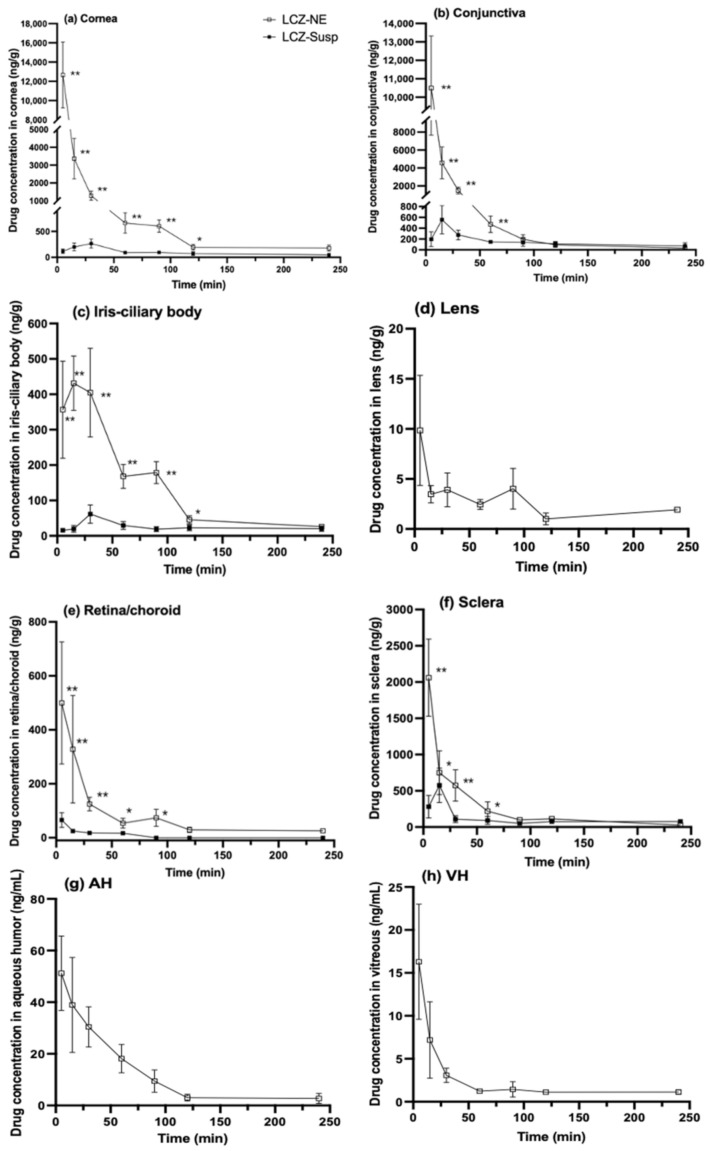
Concentration−time charts of LCZ in rabbit (**a**) cornea, (**b**) conjunctiva, (**c**) iris-ciliary body, (**d**) lens, (**e**) retina/choroid, (**f**) sclera, (**g**) AH and (**h**) VH after one topical application of the LCZ-NE and LCZ-Susp. Data represented as mean ± SD, *n* = 6; * *p* < 0.05, ** *p* < 0.01, compared to LCZ-Susp. (AH, aqueous humor; VH, vitreous humor. The drug levels in the lens, AH and VH of rabbit eyes in the LCZ-Susp group were undetectable).

The charts of the concentration-time course of LCZ concentration in the rabbit cornea, conjunctiva, iris-ciliary body, lens, retina/choroid, sclera, AH and VH samples that were collected at 5, 15, 30, 60, 90, 120 and 240 min are shown in Figure 12 after one topical administration of LCZ-NE and LCZ-Susp. The pharmacokinetic parameters in different ocular tissues are listed in Table 5. The drug concentrations in the cornea, conjunctiva, iris-ciliary body, retina/choroid and sclera tissues of rabbit eyes in the test group (LCZ-NE) were significantly higher than those in the rabbit eyes of the control group (LCZ-Susp) at the corresponding time points (*p* < 0.05). The drug levels in the lens, AH and VH of rabbit eyes that were treated with the LCZ-NE could be detected; however, those of rabbit eyes treated with the LCZ-Susp were below detection limits. The areas under the drug-concentration curve (AUC_(0–240min)_ (ng/g(mL)⋅min)) for the cornea, conjunctiva, iris-ciliary body, retina/choroid and sclera samples in rabbit eyes treated with the LCZ-NE were 9.88-, 5.55-, 5.33-, 8.63- and 2.38-fold higher, respectively, than those in the corresponding tissues in the control group. The AUC_(0–240min)_ of the lens, AH and VH in rabbit eyes treated with the LCZ-NE were 590.93 ng/g⋅min, 2776.83 ng/mL⋅min and 513.03 ng/mL⋅min, respectively. The results demonstrated that the NE as an ODDS could greatly increase the ocular bioavailability of LCZ compared to pure drug. 

Finding an effective ODDS is still a challenge for pharmaceutical scientists, due to ocular anatomical and physiological barriers, for example, tear turnover, epithelial tight junctions, reflex blinking, metabolism as well as nasolacrimal drainage. These factors make it difficult for a drug to be delivered to the target site in the eye [61]. As a consequence of these hindrances, less than 5% of topically applied drugs penetrate into deeper ocular tissues [22]. In order to effectively exert antifungal effects, drug levels should be above MIC for a certain period of time. Higher drug concentrations for shorter durations did not perform efficiently, as a result of the unavailability of drugs for at least three life cycle of microorganisms at the target site [62]. For FK, the corneal stroma which the fungi spores penetrate to proliferate is the main target site of antifungal agents. It is critical for an FK treatment to successfully deliver enough drugs into the corneal stroma [63]. The LCZ levels in the cornea were all observed to be above the MIC during the test period after a single dose of the LCZ-NE was applied, suggesting that it could maintain an effective therapeutic level for at least four hours. Thus, it was concluded that the LCZ-NE could maintain the drug level above the MIC for a sufficiently long period for better efficacy and good patient compliance. Moreover, drug delivery via topical administration to the ocular posterior segment is still a tough challenge. The successful treatment of FK and related endophthalmitis, or other intraocular fungal infections, requires long-term drug delivery. This study on NE-loaded LCZ demonstrated encouraging results that the drug could penetrate into intraocular tissues after a single topical application to rabbits. Results indicate that the cornea, conjunctiva, AH, lens, iris-ciliary body, sclera, VH and retina/choroid tissues retained the drug at concentrations of 177.12 ± 58.72 ng/g, 30.93 ± 9.64 ng/g, 2.75 ± 1.96 ng/mL, 1.92 ± 0.16 ng/g, 26.19 ± 4.98 ng/g, 26.82 ± 11.2 ng/g, 1.13 ± 0.26 ng/mL and 25.64 ± 7.78 ng/g after one single dose of LCZ-NE for 240 min, respectively, which were all above the MIC_90_ against fungi.

A previously published paper showed that absorption of drugs mainly occurred via corneal and non-corneal pathways with doses topically administered to the ocular surface [64]. Penetration across the cornea is considered to be the main route, and the cornea plays a key role as a protective barrier on the ocular surface. Thus, drugs that are absorbed into the cornea may enter the AH and other intraocular tissues, suggesting that there is good access for the delivery of drugs to the posterior segment of the eye. Transscleral drug delivery, especially conjunctival-scleral, is another important pathway to effectively deliver hydrophilic drug/nanoparticles into the posterior segments [65]. In this study, the C_max_ values for LCZ into the cornea, conjunctiva and sclera after a single dose of the LCZ-NE were 12,657.37 ± 3418.01 ng/g, 10,497.64 ± 2822.14 ng/g and 2059.57 ± 532.24 ng/g, respectively, vs. 265.91 ± 85.88 ng/g, 555.75 ± 261.80 ng/g and 281.71 ± 154.64 ng/g for the LCZ-Susp, respectively. NEs may facilitate LCZ to penetrate both the corneal and conjunctiva-sclera pathways. The C_max_ values for LCZ into the AH, VH and lens after a single dose of the LCZ-NE were 51.23 ± 14.4 ng/mL, 16.29 ± 6.7 ng/mL and 9.85 ± 5.5 ng/g, respectively, which constituted hydrophilic liquid or tissues, much lower than in the iris-ciliary body and retina/choroid tissues (C_max_ values were 431.56 ± 76.88 ng/g and 499.76 ± 226.19 ng/g, respectively) which were lipophilic tissues. This could be attributed to the high lipophilicity of LCZ. 

## 4. Conclusions

In this study, LCZ became successfully entrapped in the lipidic core, providing a nano-sized ocular delivery system. The formulation was optimized by CCD-RSM. To the best of our knowledge, this is the first in vivo investigation on the ocular biodistribution and pharmacokinetics using LCZ loaded in nano-lipid carriers. The optimized LCZ-NE has very small size, PDI and a high EE of LCZ, with at least two months of stability. The LCZ-NE showed enhanced in vitro antifungal activity and pharmacodynamic activity compared to pure drug suspension. The results of the ocular biodistribution of the LCZ-NE demonstrated that the levels of LCZ in the cornea were significantly higher in the LCZ-NE group than in the LCZ-Susp group. The LCZ-NE could penetrate across the intact cornea as well as the conjunctiva to reach the sclera, iris-ciliary body, AH, lens, VH and retina/choroid. Since the LCZ-NE exhibited good penetration across the intact cornea and conjunctiva to reach the inner eye tissues at levels above the MIC_90_, it could become an alternative for the treatment of ocular fungal infections. During treatment for FK, when cornea is healed, it is critical for a drug to penetrate the nearly intact cornea in order to reach inner ocular tissues, and to prevent relapse of infection. In order to further evaluate the therapeutic effects of the LCZ-NE for eye treatments, it is also necessary to evaluate its ocular efficacy in FK models in vivo. In summary, the LCZ-NE system exhibited an enhanced ability to penetrate the intact cornea and conjunctiva, and subsequently provide drug levels significantly higher than suspensions in the intraocular tissues, suggesting that it could be a potential candidate for the treatment of endophthalmitis, in addition to keratitis, during the course of therapy.

## Figures and Tables

**Table 1:** The results of central composite design.

Run	X_1_ (Weight of Oil, g)	X_2_ (Km)	Y_1_ (Droplet Size, nm)	Y_2_ (Drug Content, mg/mL)
1	0.13	3.56	13.13	1.34
2	0.2	2.5	15.90	1.74
3	0.1	2.5	12.99	1.20
4	0.13	1.44	14.47	1.32
5	0.27	3.56	17.47	2.33
6	0.2	2.5	15.94	1.76
7	0.2	1	18.40	1.68
8	0.27	1.44	21.08	2.58
9	0.3	2.5	20.05	2.79
10	0.2	4	14.97	1.81
11	0.2	2.5	15.99	1.81
12	0.2	2.5	16.13	1.81
13	0.2	2.5	16.09	1.85

**Table 2:** Physicochemical properties of the optimized LCZ-NE.

DS (nm)	PDI	ZP (mV)	pH	EE (%)
18.43 ± 0.05	0.070 ± 0.008	−0.202 ± 0.081	6.22 ± 0.04	98.37 ± 0.47

Note: DS, droplet size; PDI, polydispersity index; ZP, zeta potential; EE, entrapment efficiency.

**Table 3:** The squared correlation coefficients (*R*^2^) and equations for the different mathematical models fitted to the release of LCZ.

	LCZ-NE		LCZ-Susp	
	Equation	*R* ^2^	Equation	*R* ^2^
Zero order	Q=0.717× t + 26.143	0.6396	Q=0.334× t + 6.453	0.9144
First order	Q=73.475×$\;(1-e^{-0.102t}$)	0.9891	Q=33.260×$\;(1-e^{-0.036t}$)	0.9016
Higuchi	Q=7.450× t^1/2^ + 15.850	0.9671	Q=3.532× t^1/2^ + 0.460	0.9976
Korsmeyer–Peppas	Q=17.771× t^0.350^	0.9040	Q=3.758× t^0.489^	0.9718

**Table 4:** Antifungal activity of the LCZ-NE and LCZ-Susp against *Fusarium* and *Aspergillus*.

Tested Isolate	LCZ-NE		LCZ-Susp	
	Diameter (mm)	MIC_90_ (ng/mL)	Diameter (mm)	MIC_90_ (ng/mL)
Fusarium	24.8 ± 1.10	10	/	23
Aspergillus	35.4 ± 1.14 ^a^	2	19.8 ± 0.84	5

Note: ^a^ *p* < 0.01, compared to LCZ-Susp (*n* = 5).

**Table 5:** Pharmacokinetic parameters of LCZ in rabbit eyes following one topical application of the LCZ-NE and LCZ-Susp.

Tissue	Drug	Pharmacokinetic Parameters				
		C_max_ (ng/g (mL))	T_max_ (Min)	t_1/2_ (Min)	${\mathrm{AUC}}_{\left(0–240\min\right)}\;(\mathrm{ng}/g\;(\mathrm{mL})\mathrm{Min})$	${\mathrm{AUC}}_{\left(0-\infty \right)}\;(\mathrm{ng}/g\;(\mathrm{mL})\mathrm{Min})$
Cornea	LCZ-NE	12,657.37 ± 3418.01	5	33.05	228,838.63	229,578.03
	LCZ-Susp	265.91 ± 85.88	30	202.07	23,151.19	37240.62
Conjunctiva	LCZ-NE	10,497.64 ± 2822.14	5	25.67	198,353.65	198,486.26
	LCZ-Susp	555.75 ± 261.80	15	66.64	35,729.78	38,657.49
Iris-ciliary body	LCZ-NE	431.56 ± 76.88	15	53.35	32,551.25	34,554.76
	LCZ-Susp	61.79 ± 26.11	30	360.81	6110.88	16,335.28
Lens	LCZ-NE	9.85 ± 5.5	5	46.12	590.93	601.96
	LCZ-Susp	ND	ND	ND	ND	ND
Retina/choroid	LCZ-NE	499.76 ± 226.19	5	93.11	18,186.35	21,585.72
	LCZ-Susp	65.38 ± 27.29	5	51.40	2108.20	2180.50
Sclera	LCZ-NE	2059.57 ± 532.24	5	59.12	57,457.28	59,769.62
	LCZ-Susp	281.71 ± 154.64	15	181.98	26,336.85	38,856.55
AH	LCZ-NE	51.23 ± 14.4	5	62.37	2776.83	3020.87
	LCZ-Susp	ND	ND	ND	ND	ND
VH	LCZ-NE	16.29 ± 6.7	5	60.76	513.03	537.12
	LCZ-Susp	ND	ND	ND	ND	ND

Note: AH, aqueous humor; VH, vitreous humor. LCZ levels in the lens, AH and VH of rabbit eyes in the LCZ-Susp group were undetectable (ND: not detected).

## Data Availability

The data presented in this study are available on request from the corresponding author.
